# Effect of Surface Dissolution on Dislocation Activation in Stressed FeSi6.5 Steel

**DOI:** 10.3390/ma15217434

**Published:** 2022-10-23

**Authors:** Dong Zhao, Feng Ye, Binbin Liu, Haoyang Du, Yaakov B. Unigovski, Emmanuel M. Gutman, Roni Shneck

**Affiliations:** 1 State Key Laboratory for Advanced Metals and Materials, University of Science and Technology Beijing, 30 Xueyuan Road, Beijing 100083, China; 2 State Grid Jilin Electric Power Research Institute, No. 4433 Renmin Street, Changchun 130021, China; 3 Department of Materials Engineering, Ben-Gurion University of the Negev, Beer-Sheva 84105, Israel

**Keywords:** stress relaxation rate, internal stress, thermal activation energy, corrosive medium

## Abstract

The effects of surface dissolution on dislocation activation in FeSi6.5 steel are quantitatively studied by analyzing the stress relaxation data using the thermal activation theory of dislocation. The stressed FeSi6.5 steel sample in acid solutions (H_2_SO_4_ or HCl) exhibits a much higher rate of stress reduction with time compared with that in air or deionized water. As the stress relaxation time is prolonged to 20 min, the relaxation rates are 0.055 MPa·min^−1^ in water and 0.074, 0.1, 0.11 MPa·min^−1^ in H_2_SO_4_ solutions with pH 4, 3, and 2, respectively. In a NaCl solution, a slight increase in the relaxation rate compared with air was found. Higher acidity (lower pH) of the solution inducing higher stress relaxation rate implies the softening is associated with the anodic dissolution of the surface layer and the accelerated (additional) flow of dislocations. The analyses using the thermal activation theory of dislocation during relaxation reveal the mechanism for the accelerated plastic flow induced by the corrosive medium. The variations of these parameters are related to the relaxation of the stress field of dislocations and the weakening of interaction between slip dislocations and short-range obstacles. The chemomechanical effect, including a reduction in apparent activation energy and a decrease in waiting time for dislocation to obtain sufficient thermal activation energy to cross obstacles, causes an increase in the stress relaxation rate (plastic strain rate). The study confirms that surface dissolution accelerates the plastic flow of metals and supports the view that surface dissolution facilitates dislocation slip. It is helpful to improve the formability of brittle metals.

## 1. Introduction

People have found that surface dissolution could result in increased creep rate of cadmium and zinc single crystals [1], copper [2], iron and steel [3], magnesium and aluminum alloys [4,5], accelerated stress relaxation of magnesium and magnesium alloys [6,7] and decreased deformation resistance of 25Cr-60Ni-15V alloy [8], 1020 steel, 1070 steel, and FeSi6.5 steel [9]. The phenomena were of great scientific interest because the mechanical properties of a stressed metal or alloy present a high sensitivity to surface atom dissolution caused by corrosive medium or anodic polarization. The practical significance behind this chemomechanical effect [9] also attracted much attention. It has shown advantages in the surface treatment of steels [8] and preparations of wires [10,11] and rods [12] for difficult-to-deform alloys. However, its operating mechanism is still poorly understood.

On the surface of a stressed metal in a dissolved state, there were at least two irreversible processes involved, the plastic flow and the electrochemical dissolution, interacting with each other. Experimental observations and theoretical calculations [13] proposed that the changes in the mobility of dislocation and the interaction between the dislocations and obstacles in dissolution conditions correspond to the variations in plastic deformation and mechanical properties. Many cases showed that the chemomechanical effect enhanced the formability and reduced the strength of the metals. Uhlig [3] and his colleague [2] indicated that vacancies, divacancies, and dislocations resulting from the surface dissolution injected into the metal lattice and promoted dislocation climbing and slipping. It was also proposed that the accelerated flow was attributed to the removal of an oxide film or ‘debris layers’ in a corrosive solution, which reduced the resistance of the exit of dislocation from the surface [14]. Van Der Wekken [15] indicated that the thinning diameter caused by the dissolution may be responsible for the increasing flow rate. However, a general view supported that this phenomenon resulted from the synergistic action of mechanical and electrochemical factors [13]. Further understanding of the mechanism could be acquired by investigating the effect of surface dissolution on the thermal activation behavior of dislocation in polycrystals.

Stress relaxation can be used to analyze thermal activation behaviors of dislocation [16]. A typical stress relaxation test is performed by interrupting a uniaxial tensile test in the uniform elongation region at a constant total strain for a well-defined time interval. The sample continues to be deformed plastically at the expense of the reduction in the elastic strains in the machine elements as well as in the sample. This leads to a reduction in the applied stress with time since the total strain *ε*_t_ remains invariant (*ε*_t_ = *ε*_e_ + *ε*_p_ = *C*, *ε*_e_ and *ε*_p_ are the elastic and plastic components of the strain, respectively) during relaxation, *dε*_t_/*dt* = 0.

(1) $$-\dot{\sigma }=-E^{\prime }{\dot{\varepsilon }}_{e}=E^{\prime }{\dot{\varepsilon }}_{p}$$

where σ ˙, ${\dot{\varepsilon }}_{e},$ and $\dot{\varepsilon_{p}}$ are stress relaxation rate, the elastic strain rate, and the plastic strain rate, respectively, the *E*′ is the combined modulus of the specimen–machine system.

According to the Orowan equation [17], plastic strain rate depends on mobile dislocation density and average dislocation velocity.

(2) $${\dot{\varepsilon }}_{p}=\varphi \rho_{m}b\bar{\nu }$$

where *φ* is a constant, *ρ_m_* mobile dislocation density, *b* Burgers vector, and $\bar{\nu }$ average dislocation velocity.

During stress relaxation, the plastic strain caused by stress relaxation is small (<0.1%), and *ρ*_m_ and internal stress *σ*_i_ are considered to be approximately constant. Therefore, $\dot{\varepsilon_{p}}$ mainly depends on $\bar{\nu }$. It can be written in the Arrhenius form [18]

(3) $$\bar{\nu }=\nu_{0}\exp\left[-\frac{\Delta G}{kT}\right]$$

where *ν*_0_ is the pre-exponential factor, which is related to the average distance covered by the dislocation in every activation act, the frequency, and the geometric factors. *k* is the Boltzmann constant, and *T* is the absolute temperature.

The transition from elastic strain to plastic strain is the result of the activation of dislocations by surmounting obstacles under the combined action of thermal vibration and effect stress σ^*^ (acting on the dislocation line by external stress to overcome internal stress, $\sigma^{*}=\sigma -\sigma_{i}$). The apparent activation energy Δ*G* may be approximated by a simple relation [18]

(4) $$\Delta G=\Delta G_{0}-v^{*}\sigma^{*}=\Delta G_{0}-v^{*}\left(\sigma -\sigma_{i}\right)$$

where Δ*G*_0_ is the barrier activation energy at zero stress, v^*^ is the apparent activation volume, and the v^*^σ^*^ is mechanical work supplied by effective stress.

By combining Equations (1)–(4), we have

(5) $$-\dot{\sigma }=E^{\prime }\varphi \rho_{m}b\nu_{0}\exp\left[-\frac{\Delta G_{0}-v^{*}\sigma^{*}}{kT}\right]$$

From the integration of Equation (5), a model of stress relaxation is obtained as the following logarithmic law:

(6) $$\Delta \sigma =\sigma_{\left(t\right)}-\sigma_{\left(0\right)}=-\alpha ln\left(\beta t+1\right)$$

where *t* is the relaxation time, *σ*_(0)_ is the stress at the beginning of the relaxation (*t* = 0), and *α* and *β* are constants.

$$\alpha =\frac{kT}{v^{*}},\;\beta =\frac{E^{\prime }\varphi \rho_{m}b\nu_{0}v^{*}}{kT}\exp\left[-\frac{\Delta G_{0}-v^{*}\sigma_{\left(0\right)}^{*}}{kT}\right]$$

Stress relaxation of metals is essentially a thermally activated plastic flow. A relaxation curve can provide information about the activation behavior of dislocation. The activation parameters (*σ*_i_, v^*^ and Δ*G*_0_) can be obtained by analyzing stress relaxation data using the model of stress relaxation. These parameters characterize the plastic deformation mechanism. Wang et al. [19] obtained the v^*^ and the Δ*G*_0_ of Fe-based alloys in hydrogen-charged and hydrogen-free conditions using a stress relaxation test and indicated that the lower values of the v^*^ and the Δ*G*_0_ in hydrogen charged were related to the decrease in the interaction between glide and forest dislocations and the reduction in the resistance of dislocation motion due to hydrogen shielding effect. Trojanová et al. [20] estimated the *σ_i_* at various temperatures for AZ63 magnesium alloy by analyzing the stress relaxation curve and indicated that the nearly constant level of the $\sigma_{i}$ at 200 and 300 °C was related to dynamic recovery caused by dislocation climb and the activity in additional non-basal slip systems. The investigation of stress relaxation was helpful in revealing dislocation processes responsible for plastic deformation.

The purpose of the present study is to understand the dislocation migration mechanism of the chemomechanical effect using the thermal activation theory of dislocation during relaxation and to provide a reference for improving the formability of difficult-to-deform metals. FeSi6.5 steel is known as an excellent soft magnetic material with high electrical resistance, high permeability, and nearly zero magnetostriction [21,22]. However, it is difficult to deform at room temperature, which prevents its industrial application [23]. Our previous results showed that the anodic polarization on FeSi6.5 steel can relieve its work hardening and improve its formability [11]. In the present work, it was observed that stress relaxation rates for the FeSi6.5 steel increased significantly with the acidity. The analyses suggest that the variation in the dislocation mobility resulting from the chemomechanical effect causes the increase in the plastic strain rate. The study confirms that surface dissolution accelerates plastic flow of metals and support the view that surface dissolution facilitates dislocation slip. It implies that the significant difficulties at plastic deformation of metals and alloys with low or limited ductility can be overcome by using a special highly efficient electrolytic active medium.

## 2. Materials and Methods

The material selected for this work was FeSi6.5 steel in the form of a wire with a diameter of 2 mm at hot-drawn conditions. The FeSi6.5 steel was prepared by vacuum induction casting, rotary swaging, and hot drawing. The details about the processing methods of FeSi6.5 steel samples can be found elsewhere [24]. Its chemical compositions are listed in Table 1. Prior to tests, the oxide layer on the wire’s surface was removed with silicon carbide abrasive paper. Specimens with a length of 200 mm were cut from the wires.

Similar to our previous tensile test in H_2_SO_4_ solution [11], the stress relaxation test in a corrosive medium was performed on an electronic tensile test machine (CMT5105) equipped with a plastic cylindrical tank with a volume of 700 mL. The wire sample passes through the cell with the center part of 100 mm in length, immersing in the medium. In addition, in order to maintain a constant temperature (20 °C) and pH value of the medium during the test, another large constant temperature tank was connected to the plastic circular tank. The medium was kept circulating between the two tanks through a pump. The mediums used in this work involved deionized water (pH = 7, as a reference), NaCl solution (pH = 7), NaCl + HCl solution (pH = 1) and H_2_SO_4_ solutions (pH = 2, 3, and 4). Both ends of the specimen were fixed in a crosshead holder. After the specimens were deformed to the preset stress value at a strain rate of 10^−4^ s^−1^, the crosshead stopped to maintain a constant level of strain, and the stress was monitored as a function of time.

The microstructure of the samples was examined using a Zeiss Supra 55 scanning electron microscope (SEM). A JEM-2100 transmission electron microscopy (TEM) was used to detect the dislocation configuration by operating at 200 kV. The specimens for TEM were mechanically thinned down to 50 μm in thickness before being electropolished in a solution consisting of 5 wt% perchloric acid and 95 wt% ethanol at −20 °C with a voltage of 30 V.

## 3. Results

**Figure 1:**
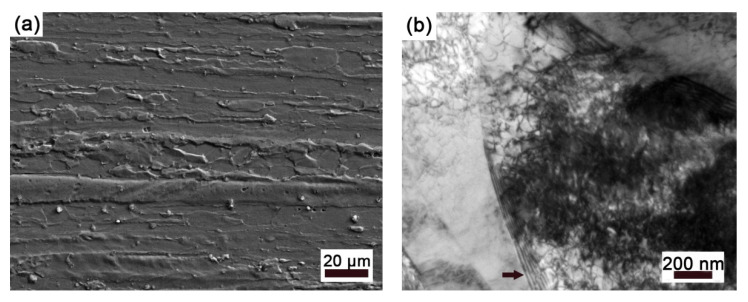
(**a**) SEM micrograph of microstructure and (**b**) TEM micrograph of dislocation morphology in grain for hot-drawn sample.

Figure 1a shows the SEM micrograph of the deformation structure along the longitudinal section in the hot-drawn specimen. It presents typical hot drawing characteristics with strip grains along the drawing direction and small block grains. In grains, it is found that some dislocations are piled up on grain boundary (arrow), while others present a tangle characteristic (Figure 1b).

**Figure 2:**
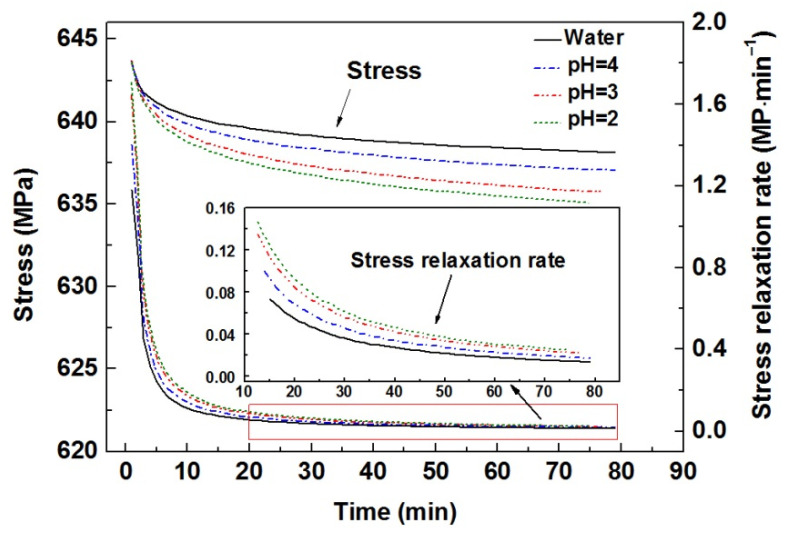
The variations of stress and relaxation rate versus time for samples immersed in water and H_2_SO_4_ solutions with pH 4, 3, and 2, respectively, during relaxation.

Figure 2 shows stress relaxation behaviors for FeSi6.5 steel samples in water and H_2_SO_4_ solutions with various pH values in terms of changes in stress and relaxation rate versus time. The same initial stress was applied to all the samples. As evident from the curves, for all the cases, the stresses and the relaxation rates decrease with time. The relaxation rates present a two-stage characteristic, the higher values at the initial several minutes followed by the lower values for most of the time. The stress relaxation rates for samples in H_2_SO_4_ solution are higher than that of samples in water. The result is similar to the decrease in the drawing force of Mg alloy [12] and the reduction in the hardness of pure iron [25] when samples were subjected to anodic polarization in an H_2_SO_4_ solution. One can see that the relaxation rates increase with the solution pH value decreasing. For example, as the stress relaxation time is prolonged to 20 min, the relaxation rates are 0.055 MPa·min^−1^ in water and 0.074, 0.1, and 0.11 MPa·min^−1^ in H_2_SO_4_ solutions with pH 4, 3, and 2, respectively.

**Figure 3:**
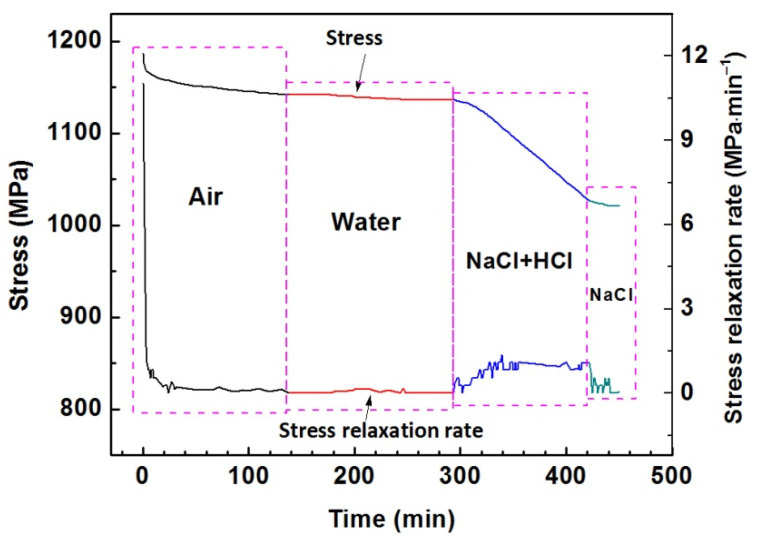
The variations of the stress and the relaxation rate versus time for FeSi6.5 steel sample in successive relaxation in air, water, HCl + NaCl solution, and NaCl solution.

A stressed sample relaxed successively in air, water, HCl + NaCl solution, and NaCl solution. Figure 3 shows the variations of the stress and the stress relaxation rate with time during successive relaxation. The sample was first loaded to 1187 MPa at a strain rate of 1 × 10^−4^ s^−1^ and relaxed in air until the applied stress trend a constant value. Water was slowly injected into the plastic cell at first. The liquid in the plastic cell was totally replaced by HCl + NaCl and NaCl solution in sequence with a relaxation time of 60 or 30 min (Figure 3).

The applied stress drops slowly and trends a constant value after relaxation for 138 min in air. As water was injected into the plastic cell, nearly no change occurred in the relaxation rate of the specimen. The stress accumulatively dropped down only 5 MPa in 155 min, with a very low relaxation rate of 0.032 MPa·min^−1^. However, after replacing the water with HCl+NaCl solution, the relaxation rate first increases slowly for a few minutes, reaching a higher value of 1.1 MPa·min^−1^. The stress drops down by 110.4 MPa in 129 min. After replacing the HCl+NaCl solution with NaCl solution, the relaxation rate decreases to 0.27 MPa·min^−1^ and accumulatively reduces by 5.6 MPa in 28 min.

## 4. Discussion

The plastic flow of metals during stress relaxation is related to the thermal activation of dislocation. The activation parameters (*σ*_i_, v^*^, and Δ*G*_0_) are obtained by analyzing stress relaxation data [26,27]. The effect of surface dissolution on dislocation activation is investigated by comparing the differences in the activation parameters in water and corrosive mediums.

### 4.1. The Internal Stresses

The internal stress is determined by analyzing stress relaxation data using Li’s method [18].

According to Johnston and Gilman [28], the average velocity of dislocations is related to the effective stress applied to the dislocation by the following relation.

(7) $$\bar{\nu }=B{\left(\sigma -\sigma_{i}\right)}^{m^{*}}$$

where *B* is the average velocity at unit effective stress and m* is the dislocation velocity-stress exponent.

By combining Equations (1), (2), and (7) and integrating the relation obtained, the following is derived:

(8) $$\sigma -\sigma_{i}=k{\left(t+\tau \right)}^{-n}$$

where $k={\left[\left(m^{*}-1\right)E^{\prime }\varphi \rho_{m}bB\right]}^{-n}$, $n=\frac{1}{m^{*}-1}$, and *τ* is the integration constant.

Taking the logarithm of Equation (8), we have

(9) $$\ln\left(-\dot{\sigma }\right)=\ln\left(nk\right)-\left(n+1\right)\ln\left(t+\tau \right)$$

If the relaxation curve is adequately described by Equation (9), the plot of $\ln\left(-\dot{\sigma }\right)$ against ln(t+τ) is linear (for large *t*), and the slope of the curve gives m*. The constant *τ* can be obtained by the deviation from linearity at a short time. The internal stress *σ*_i_ can be estimated from the following relation [18].

(10) $$\frac{\sigma_{1}-\sigma_{i}}{\sigma_{2}-\sigma_{i}}={\left(\frac{t_{1}+\tau }{t_{2}+\tau }\right)}^{-1/\left(m^{*}-1\right)}$$

Here, *σ*_1_ and *σ*_2_ are stresses at times *t*_1_ and *t*_2_ during relaxation, respectively.

**Figure 4:**
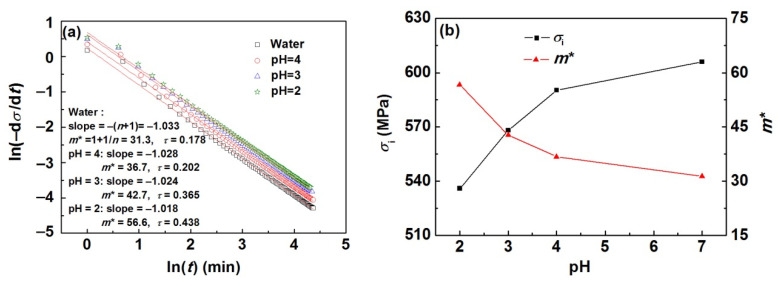
(**a**) Log-log plot of $\dot{\sigma }$ vs. *t* in water and H_2_SO_4_ solutions with various pH values, (**b**) variations of *σ*_i_ and m* with pH values.

As shown in Figure 4a,b, for all the cases, the plots of $\ln\left(-\dot{\sigma }\right)$ versus ln(t) are linear for a long time, and the obtained values of m* and *τ* increase with the pH value decreasing. The internal stresses *σ*_i_ calculated using the values of m* and *τ* are found to be 606.0 MPa in water as well as 590.2, 568.2, and 535.9 MPa in H_2_SO_4_ solutions with pH values of 4, 3, and 2, respectively, and present a decreasing trend with the decrease in pH values.

### 4.2. Thermal Activation of Dislocation

Using the stress relaxation data, the apparent activation volume v^*^ can be determined by Equation (6). Plotting Δ*σ* against ln(*t*), a straight line with the slope of *kT*/v^*^ is obtained.

The v^*^ is also obtained by the following relation from taking the logarithm of Equation (5)

(11) $$\ln\left(-\dot{\sigma }\right)=\ln\left(E^{\prime }\varphi \rho_{m}b\nu_{0}\right)-\frac{\Delta G_{0}}{kT}+\frac{v^{*}\sigma^{*}}{kT}$$

Plotting $\ln\left(-\dot{\sigma }\right)$ against σ^*^, gives a straight line with the slope of v^*^/*kT*.

**Figure 5:**
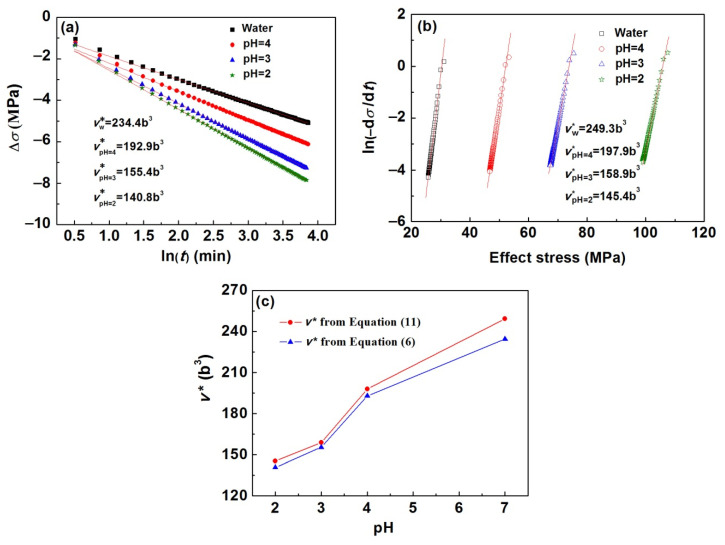
Fitting of the experimental stress relaxation data for FeSi6.5 steel samples in water and H_2_SO_4_ solution using Equation (6) (**a**) and Equation (11) (**b**) and the obtained activation volumes are shown as a function of pH values (**c**).

As illustrated in Figure 5a,b, the values of v^*^ obtained using Equations (6) and (11) are 234.4, 192.9, 155.4, and 140.8 b^3^, as well as 249.3, 197.9, 158.9, and 145.4 b^3^ for samples immersed in water and H_2_SO_4_ solutions with pH values of 4, 3, and 2, respectively. It can be seen that the values of v^*^ obtained by the two methods are very close, and all present a decreasing trend with the decrease in pH values (Figure 5c).

In fact, the meaningful quantity to characterize the obstacle to dislocation motion is the Δ*G*_0_, the Gibbs free enthalpy necessary for overcoming a short-range obstacle without the stress.

A method for determining Δ*G*_0_ based on Equation (11) was proposed in Ref. [18]. Plotting $\ln\left(-\dot{\sigma }\right)$ versus σ^*^ and extrapolating the straight line to σ^*^ = 0 gives the segment $\delta =\ln\left(E^{\prime }\varphi \rho_{m}bv_{0}\right)-\frac{\Delta G_{0}}{kT}$. Therefore Δ*G*_0_ can be derived from the segments for at least two different temperatures. Regretfully, in the present work, the test temperature is limited to less than 100 °C. The Δ*G*_0_ cannot be obtained by the method.

The method proposed in Ref. [19] is used for estimating the variation of Δ*G*_0_ caused by surface dissolution. From Equation (5), the Δ*G*_0_ of samples in water and H_2_SO_4_ solutions can be expressed as

(12) $$\Delta G_{0\left(w\right)}=-kT\ln\left(\frac{-\dot{\sigma_{w}}}{E^{\prime }\varphi \rho_{m\left(w\right)}bv_{0}}\right)+v_{w}^{*}\sigma_{w}^{*}$$

(13) $$\Delta G_{0\left(s\right)}=-kT\ln\left(\frac{-\dot{\sigma_{s}}}{E^{\prime }\varphi \rho_{m\left(s\right)}bv_{0}}\right)+v_{s}^{*}\sigma_{s}^{*}$$

where *E*′, *φ*, *b*, and *v*_0_ maintain constant in water and H_2_SO_4_ solution. The parameters with subscripts w and s in the formulas are the corresponding parameters in water and H_2_SO_4_ solution, respectively.

From Equations (12) and (13), the variation of Δ*G*_0_ caused by surface dissolution, Δ*G*_0(sw)_, expressed as

(14) $$\Delta G_{0\left(\mathrm{sw}\right)}=\Delta G_{0\left(s\right)}-\Delta G_{0\left(w\right)}=kT\ln\left(\frac{\dot{\sigma_{w}}}{\dot{\sigma_{s}}}\cdot \frac{\rho_{m\left(s\right)}}{\rho_{m\left(w\right)}}\right)+v_{s}^{*}\sigma_{s}^{*}-v_{w}^{*}\sigma_{w}^{*}$$

The Δ*G*_0(sw)_ at relaxation time *t* = 25 min is estimated as an example. The values of $\dot{\sigma_{w}}$, $\dot{\sigma_{s}}$, $v_{s}^{*}$, $\sigma_{s}^{*}$, $v_{w}^{*}$, and $\sigma_{w}^{*}$ are obtained in the above results. The mobile dislocation density *ρ*_m_ is a poorly documented parameter. Indeed, it is frequently assumed to be a constant fraction of the total dislocation density *ρ* [29]. In this work, the value of $\frac{\rho_{m\left(s\right)}}{\rho_{m\left(w\right)}}$ is estimated according to the value of $\frac{\rho_{s}}{\rho_{w}}$. The estimated value may be slightly lower than the actual value as surface dissolution can increase the mobility of dislocation. The values of $\frac{\rho_{s}}{\rho_{w}}$ are calculated by the relation between internal stress and dislocation density ($\sigma \propto \rho^{1/2}$). In the end, the values of the Δ*G*_0(sw)_ at relaxation time t = 25 min is estimated to be −0.011, −0.015 and −0.020 eV for H_2_SO_4_ solutions with pH values of 4, 3, and 2, respectively. Thus surface dissolution causes a slight decrease in the Δ*G*_0_.

### 4.3. Enhancing Plastic Flow with Surface Dissolution

In addition to the mobile dislocation density, another primary factor determining the plastic-flow characteristics of metals is the dislocation velocity. The greater values of $\dot{\sigma }$, σ^*^, and m^*^ in H_2_SO_4_ solution with decreasing pH value signify that the surface dissolution increases the dislocation velocity in the FeSi6.5 steel (Equation (7)). This is attributed to the reduction in the apparent activation energy caused by surface dissolution. The lower values of *σ*_i_ in H_2_SO_4_ solution signify that surface dissolution decreases the internal stress and increases the effect of stress acting on dislocations, providing more mechanical works (v^*^σ^*^) to dislocations. The v^*^σ^*^ in water and H_2_SO_4_ solution with pH values of 4, 3, and 2 at *t* = 10 min are 0.84 as well as 0.89, 1.01, and 1.34 eV, respectively. Therefore, the thermal activation energy Δ*G* required for dislocations to cross the barrier is reduced. On the other hand, the lower values of the barrier activation energy at zero stress Δ*G*_0_ in H_2_SO_4_ solution indicate that surface dissolution simultaneously decreases the resistance of short-range barriers. This is in agreement with the decrease in surface dissolution on yield strength [11]. The thermal activation energy is further decreased. The reduction in the Δ*G* leads to a shorter wait time for dislocation to obtain thermal activation energy to cross obstacles; thus, the dislocation velocity presents a higher value in the H_2_SO_4_ solution.

The effects of surface dissolution on the plastic flow and the activation of dislocation are clearly demonstrated in the successive relaxation (Figure 3). At the end of relaxation in the air, the external stress drops extremely slowly and almost trends a constant value, which implies that the external stress is close to the internal stress level as it cannot be smaller than the internal one, and the effective stress is the condition to ensure the dislocation to move forward. In this case, there is little mechanical work provided by the effective stress. The dislocation needs to wait a longer time to obtain more thermal activation energy to cross obstacles; thus, the strain rate or stress relaxation rate presents a lower value.

As water injects into the plastic tank, it is expected that the stress relaxation rate has little change as it is absent in the chemical reaction of FeSi6.5 steel in water. When the sample is subjected to HCl + NaCl solution, it is observed that the external stress drops rapidly, and it can be inferred that the internal stress will inevitably decrease. The significant decrease in the external stress in HCl + NaCl solution implies the reduction in the activation energy and the increase in the amount of activation dislocations.

**Figure 6:**
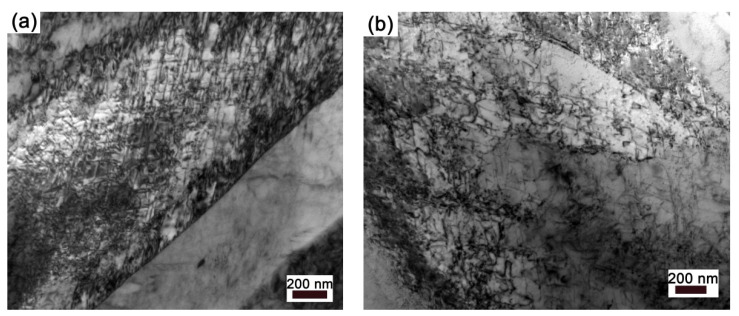
TEM images of samples deformed in air (**a**) and in H_2_SO_4_ solution (**b**).

The enhancing plastic flow and dislocation activation caused by surface dissolution can be conceived as such an operation process. Vacancies or divacancies created by selective dissolution are attracted to metallic crystal lattices [30,31]. This may cause the attenuation of inter-atomic bonds and the relaxation of the stress field provoked by dislocations [25] or bring about a chemical potential gradient for the dislocations in the surface layer [13]. These changes create favorable conditions for dislocation to pass obstacles and lead to the reduction in thermal activation energy of dislocation and the decrease in the resistance of dislocation slip. Figure 6a,b shows dislocation morphology for FeSi6.5 steel samples stretched in air and in H_2_SO_4_ solution. It is found that the dislocations in the sample deformed in air are mainly tangled structures (Figure 6a); however, that of the sample deformed in H_2_SO_4_ solution presents a relaxed structure (Figure 6b). This result indicates that surface dissolution promotes dislocation slip. The accelerated effect is related to the generation rate and the diffuse rate of the vacancies. The dependence of stress relaxation rate and activation parameters on pH value confirms this.

At the end of the article, we discuss the changes in internal stress and mobile dislocation density in the process of stress relaxation. In the above models, internal stress and density of mobile dislocations are considered constant during relaxation. The assumption seems to be justified since the plastic strain induced by stress relaxation is small and is obeyed by many metals [32]. However, recently, the changes in *σ*_i_ and *ρ*_m_ during stress relaxation were found in some materials [33,34].

In the present work, the increase in *σ*_i_ during relaxation $\Delta \sigma_{i}$ is measured. The increase in plastic strain during relaxation can be calculated using the relation, $\Delta \varepsilon_{p}=\Delta \sigma /E^{\prime }$, Δ*σ* is the drop of the applied stress. From relaxation curves (Figure 2), the Δ*σ* in 60 min is 5.1, 6.1, 7.3, and 7.8 MPa in water and H_2_SO_4_ solutions with pH values of 4, 3, and 2, respectively. The *E*′ is estimated to be 122,870 MPa in terms of tensile curves in our previous paper [11]. The increase in strain for samples in water and H_2_SO_4_ solutions with pH values of 4, 3, and 2 during stress relaxation are 4.2 × 10^−5^, 5.0 × 10^−5^, 5.9 × 10^−5^, and 6.3 × 10^−5^, respectively. The $\Delta \sigma_{i}$ caused by relaxation strain is estimated using Hollomon power law $\sigma =k\varepsilon^{n}$, *n* the strain hardening exponent, and *k* the strength coefficient. The *k* and *n* of FeSi6.5 steel are 2152, 1756, 1570, and 1414 and 0.113, 0.1, 0.1, and 0.1 in air and H_2_SO_4_ solution with electric current of 10, 15, 20, and 25 mA·cm^−2^ in our previous paper [11]. The estimated value of $\Delta \sigma_{i}$ is less than 0.012 MPa. Therefore, it is reasonable to assume that the internal stress is constant during stress relaxation.

In this work, the change of *ρ*_m_ is investigated by the method used in [34]. Taking the logarithm of Equation (6), we get

(15) $$\ln\left(-\dot{\sigma }\right)=\ln\left(\alpha \beta \right)-\ln\left(\beta t+1\right)$$

The absolute value of the slope |*S*| of $\ln\left(-\dot{\sigma }\right)$ versus ln(t) has a corresponding relationship with the trend of variation of $\frac{\rho_{m}}{\rho_{m\left(0\right)}}$ versus ln(t) (the *ρ*_m(0)_ is the initial density of mobile dislocations). If |*S*| ≈ 1, $\frac{\rho_{m}}{\rho_{m\left(0\right)}}$ versus ln(t) is linear, signifying *ρ*_m_ is constant during relaxation. If the |*S*| deviates significantly from 1, namely |*S*| > 1 or |*S*| < 1, the curve of $\frac{\rho_{m}}{\rho_{m\left(0\right)}}$ versus ln(t) concave down or concave up, respectively, signifying that the *ρ*_m_ is variable during relaxation. As shown in Figure 4, the |*S*| in water and H_2_SO_4_ solutions with various pH values are approximately equal to 1. Thus it is reasonable to assume that the density of mobile dislocations is constant during relaxation.

## 5. Conclusions

1. The stressed FeSi6.5 steel samples subjected to H_2_SO_4_, HCl + NaCl, and NaCl solutions present an increasing stress relaxation rate compared with one in air or water. The acceleration effect increases with decreasing pH value of the solution.
2. The internal stress, the activation volume, and the variation of barrier activation energy at zero stress for FeSi6.5 steel in water and H_2_SO_4_ solution are obtained by analyzing stress relaxation data, and their values decrease with the decrease in pH value of the solution.
3. The mechanism of surface dissolution enhancing plastic flow is quantitatively investigated by comparing the activation parameters in mediums and the dissolution process. The decreases of the internal stress and the zero stress activation energy induced by surface dissolution lead to the decrease in thermal activation energy of dislocation, and the increase in dislocation velocity, eventually causing the increase in stress relaxation rate or plastic flow rate.

## Figures and Tables

**Table 1:** Chemical composition of steels, wt%.

Materials	C	Mn	Si	S	P	Fe
FeSi6.5 steel	0.004	<0.1	6.5	0.003	0.004	balance

## Data Availability

The data presented in this study are available when requested.
